# An eco-friendly ultrasound approach to extracting yellow dye from *Cassia alata* flower petals: Characterization, dyeing, and antibacterial properties

**DOI:** 10.1016/j.ultsonch.2023.106519

**Published:** 2023-07-12

**Authors:** Moorthy Muruganandham, Kanagasabapathy Sivasubramanian, Palanivel Velmurugan, Subbaiah Suresh Kumar, Natarajan Arumugam, Abdulrahman I. Almansour, Raju Suresh Kumar, Sivakumar Manickam, Cheng Heng Pang, Subpiramaniyam Sivakumar

**Affiliations:** a Centre for Materials Engineering and Regenerative Medicine, Bharath Institute of Higher Education and Research, Selaiyur, Chennai, Tamil Nadu 600126, India; b Department of Chemistry, College of Science, King Saud University, P.O. Box 2455, Riyadh 11451, Saudi Arabia; c Petroleum and Chemical Engineering, Faculty of Engineering, Universiti Teknologi Brunei, Bandar Seri Begawan BE1410, Brunei Darussalam; d Department of Chemical and Environmental Engineering, University of Nottingham Ningbo China, Ningbo 315100, China; e Municipal Key Laboratory of Clean Energy Conversion Technologies, University of Nottingham Ningbo China, Ningbo 315100, China; f Department of Bioenvironmental Energy, College of Natural Resource and Life Sciences, Pusan National University, Miryang-si, Gyeongsangnam-do 50463, Republic of Korea

**Keywords:** Ultrasound, Extraction, Natural dye, *C. alata*, UV-spectrophotometer, Color fastness

## Abstract

Using natural dyes in dyeing industries becomes an alternative to synthetic dyes, which are known to contain harmful chemicals that can pose risks to the environment and human health. This study involves the extraction of yellow dye from *Cassia alata* flower petals, optimization of the extraction process using an ultrasonic bath (40 KHz and an input power of 500), ultrasonic probe (390 W, 455 W, 520 W, 585 W, and 650 W), and conventional heating (heating mantle with 30 °C, 40 °C, 50 °C, 60 °C, and 70 °C), characterization of the dye, as well as dyeing (cotton, silk, and leather) without using a mordant. The extracted yellow dye was further evaluated to determine its antibacterial activity against skin bacteria. Dye extraction optimization using UV–Visible spectrophotometric analysis revealed that the maximum yellow color in methanol extract (287 and 479 nm) was obtained at 50 °C for 45 min using ultrasonic water bath extraction, followed by the ultrasonic probe and direct heating. Based on the FTIR spectra, it is evident that O—H is present at approximately 3300 cm^−1^, while C—H stretches at around 2900 cm^−1^. A characteristic peak at 1608 cm^−1^ bears a striking similarity to anthraquinonoid-based compounds. Also, using the ultrasonic water bath dyeing technique at 50 °C for 45 min, the yellow color of cotton, silk, and leather was dyed optimally. Due to effective color removal after two washings with boiling soap liquid, the dyed cotton and silk fabric displayed good washing and rubbing fastness. Regarding antibacterial activity, the dye was highly active against all pathogens after extraction in methanol. The maximum inhibition was observed against *Pseudomonas* sp. with a MIC value of 1.56 mg/ml.

## Highlights

• Extraction of yellow dye from *Cassia alata* flower petals, and optimization.
• Ultrasonic bath, probe, and conventional heating used for yellow dye extraction.
• Characterization of the yellow dye, as well as dyeing (cotton, silk, and leather) without mordant.
• Yellow dye was further evaluated to determine its antibacterial activity against skin bacteria.

## Graphical abstract

**Figure:**
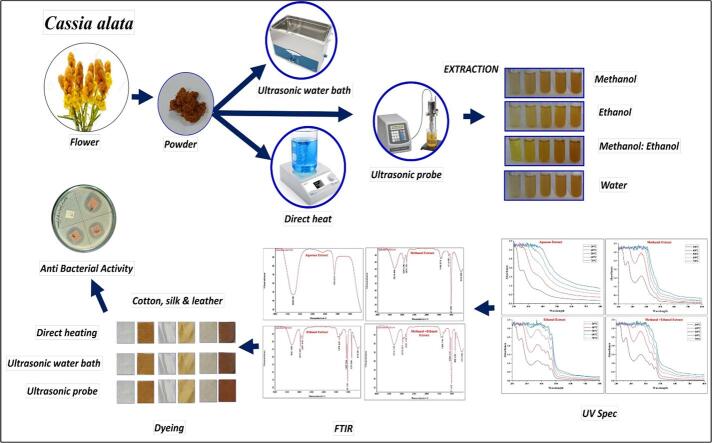

## 1 Introduction

In textile dyeing, natural or synthetic dyes add color to fibers, yarns, or fabrics. The textile industry has greatly benefited from synthetic dyes due to their advantageous characteristics and user-friendliness. However, synthetic dyes have been associated with harmful environmental and human health effects. Most artificial dyes are produced from non-renewable petroleum sources, requiring significant energy. Water generated by synthetic dyeing processes has hazardous environmental effects and poses health risks [1], [2]. The use of natural dyes has been around for thousands of years. They are environmentally friendly, renewable, and biodegradable. Their aesthetic properties are unique, and every plant-based dye has a unique chemical composition, resulting in a distinct color and shade. Natural dyes can achieve various colors, from subtle earthy tones to vibrant hues.

Furthermore, the color of natural dyes may be influenced by several factors, such as temperature, pH, and the type of fiber being dyed, making each dyeing process unique [2], [3]. As a result of environmental concerns about synthetic dyes, the fashion industry has recently turned its attention to natural dyes. Unlike synthetic dyes, natural dyes derived from plants, animals, and minerals are environmentally friendly and sustainable. In India and other parts of the world, *Cassia alata* flowers have traditionally been used to dye textiles. A member of the Fabaceae family, *C. alata* is commonly known as a candlestick plant or ringworm shrub. In the dye extracted from *C. alata* flowers, anthraquinones are present, which are natural pigments that produce various shades of yellow. This dye has good lightfastness and wash-fastness properties, meaning the color remains stable after repeated washings and exposure to sunlight [4]. When extracting heat-sensitive active chemicals, higher temperatures may harm their quality. Traditional solvent extraction methods for extracting natural products have poor extraction efficiency, more extended extraction periods, and lower yields due to the heavy usage of organic solvents [5]. By accelerating mass transfer rates and potentially rupturing cell walls due to the generation of microcavities, ultrasound can increase extraction rates, resulting in higher product yields with less solvent consumption and processing time [6], [7], [8], [9].

The range of natural dyes has significantly increased in traditional and cutting-edge application disciplines owing to investigations into novel sources of natural dyes and eco-friendly, durable, and economically viable technologies for their processing and application [10]. Natural dyes can be a good substitute for synthetic colors in an industrial setting. It can also promote sustainable practices and support local communities involved in producing natural dyes. However, the use of natural dyes in the textile industry still needs to be improved due to various challenges, such as their low colourfastness properties, variability in color, and higher cost than synthetic dyes. Optimizing natural dye extraction and dyeing processes can help overcome these challenges and promote using natural dyes in the textile industry [11].

The presence of chemical compounds, including phenols, tannins, saponins, alkaloids, steroids, flavonoids, and carbohydrates, is linked to the activity of plants. *C. alata* is one of the most important genera of Cassia species rich in anthraquinones and polyphenols. This herb *C. alata* has historically been used to treat skin diseases in humans and animals; all parts of this plant have therapeutic properties, including antibacterial properties [12]. *C. alata*, commonly known as the candlestick plant or ringworm shrub, belongs to the family Fabaceae [13]. *Cassia* flowers are a potential natural dye source traditionally used in India and other parts of the world for dyeing textiles [13]. The extracts of *C. alata* have been used in cosmetic formulations for dermatological skin care products [12].

This study focuses on extracting and optimizing natural dye from *C. alata* flowers for dyeing leather, silk, and cotton. Also, the characterization of the natural dye extracted from leather, silk, and cotton materials, its colourfastness properties and the optimization of the dyeing process parameters were investigated. The dye extracted from this plant has also been evaluated for potential use in the textile and leather industries.

## 2 Materials and methods

### 2.1 Chemicals and media

The chemicals and solvents used in this study were all analytical grade and were sourced from SRL India. The solvents methanol and ethanol used were 99.9% pure. The media was obtained from Hi-media (Mumbai, India). As a reference antibiotic, laboratory-purpose tetracycline (potency - min. 720 IU/mg) was obtained from the Hi-Media (Mumbai, India).

### 2.2 Materials collection and preparation

Flowers of *C. alata* were freshly collected near the campus of Bharath University in Chennai, Tamil Nadu, India. The petals were carefully separated from the peduncle and cut into small pieces (2 mm diameter) to facilitate extraction. These small pieces were then used to extract the dye from the petals. For further investigation, cotton (160 g m^−2^, yarn count 40 s Ne), silk (weight-79 g/sm, thread count: 99 × 102, 100% Tussar silk, plain weave), and leather (Chrome tanned wet-blue goat leather) were purchased commercially in the local market.

### 2.3 Extraction of dye using magnetic stirrer

In an extraction glass vessel, 5 g *C. alata* petals were added to 50 ml of different solvents, including double distilled water, ethanol, methanol, and ethanol + methanol (1:1), to extract the dye. The vessel was covered with aluminium foil to prevent evaporation and placed on the magnetic stirrer at 250 rpm speed and maintained at 80 °C for 60 min. A UV–Vis spectrophotometer (UV-1800, Genesys 180, Thermo Fisher Scientific, USA) was used to measure the absorbance wavelengths of dye between 200 and 800 nm for up to 60 min [4].

### 2.4 Dye extraction using ultrasound (US) water bath

In an extraction glass vessel, 5 g *C. alata* petals were added to 50 ml of different solvents, including double distilled water, ethanol, methanol, and ethanol + methanol (1:1), to extract the dye. The vessel was covered with aluminium foil to prevent evaporation and placed in the centre of a 24-litre capacity ultrasonic water bath (LABMAN, LMUC-25, India) operating at a frequency of 40 KHz and an input power of 500 W. The internal temperature of the vessel was maintained at 80 °C. The ultrasonic bath consisted of four transducers placed at the bottom of a tank with internal dimensions of 500 × 300 × 200 mm^3^. To find the best time for extracting more dye., the extraction time was continuously increased by 10 min. The samples were taken every 10 min. A UV–Vis spectrophotometer (UV-1800, Genesys 180, Thermo Fisher Scientific, USA) was used to measure the absorbance wavelengths of dye between 200 and 800 nm for up to 60 min [14].

### 2.5 Dye extraction using an ultrasonic probe

5 g *C. alata* flower petals were placed in an extraction vessel containing 50 ml of different solvents (double distilled water, ethanol, methanol, and ethanol + methanol (1:1 ratio)). In the second step, in an ultrasonic probe sonicator, the probe was placed in the middle of the vessel (LABMAN, Pro-650, India) and operated with 20 kHz frequency and different input powers of 390 W, 455 W, 520 W, 585 W, and 650 W for 30 min with a pulse on 10 sec and pulse off 3 sec. The vessel's internal temperature was increased according to the input power, and the temperature was determined with a probe attached temperature sensor. Using a UV–visible spectrophotometer (UV-1800, Genesys 180, Thermo Fisher Scientific, USA), the absorbance wavelength of dye between 200 and 800 nm for up to 60 min was measured [14].

### 2.6 Determination of energy transferred to the medium

The energy transported to the medium was investigated using the Mantas et al. [15] and Baumann et al. [16] approach with slight modifications. The volumetric density of a liquid system with ultrasound can be reported (mW·ml^−1^). The energy was calculated using Eq. (1).

(1) $$Energy\left(E\right)=MC_{p}\frac{\mathrm{dT}}{\mathrm{dt}}$$

where M is the mass (in kilogrammes), (dT/dt) is the temperature change over time (K s^−1^), and (J kg ^-1^ K^−1^) is the substances specific heat. 100 ml of SS were subjected to sonication for 3 min, with temperature readings every 5 s. The extraction medium's specific heat (1,680 J kg^−1^ K^−1^) was used for the power calculation.

### 2.7 Dye extraction using a heating mantle (direct heat)

Briefly, 50 ml of different solvents (double distilled water, ethanol, methanol, and ethanol: methanol (1:1)) and 5 g *C. alata* flower petals were added to an extraction vessel. The vessel was then placed on a heating mantle set at different temperatures (30 °C, 40 °C, 50 °C, 60 °C, and 70 °C) for 10 min. The absorbance wavelength of dye between 200 and 800 nm was measured for up to 60 min with a UV–visible spectrophotometer (UV-1800, Genesys 180, Thermo Fisher Scientific, USA) [14].

### 2.8 Parameter optimization for dye extraction

Several parameters were optimized for extracting natural dye from the petals of the *C. alata* flower. Various concentrations of yellow dye powder (1–5%) were exploited to determine the optimal concentration of the dye during extraction. In order to achieve improved outcomes, the extraction was conducted at different temperatures (between 30 °C and 70 °C at intervals of 10 °C) and for additional time (10 to 60 min). The dye colour intensity was measured using a UV–visible spectrophotometer. Optimal dye extraction conditions were selected based on the color intensity produced by the extraction process [14].

### 2.9 FTIR analysis

Fourier Transform Infrared (FTIR) spectroscopy was employed to identify the functional groups of the extracted dye (Thermo Fisher, Summit Lite, USA) in the range from 500 to 4000 cm^−1^. By cataloguing and categorizing the various vibrational modes observed in the dye, it was possible to identify specific functional groups.

### 2.10 Dyeing optimization

Cotton, silk, and leather were dyed using the extracted yellow dye using the same extraction methods, i.e., ultrasonic water bath, ultrasonic probe, and conventional heating. Before dyeing, cotton and silk materials were washed and bleached comprehensively. Similarly, the surface of the leather material was cleaned to remove the hair follicles. A constant 12% dye concentration was used for all dyeing trials. Dyeing materials were cut into 1″× 1″ square pieces. The dying conditions were optimized by adjusting the temperatures (40 °C, 50 °C, 60 °C, 70 °C, and 80 °C) for 30 min in an ultrasonic bath (40 kHz). An ultrasonic probe was utilized at different powers (390 W, 455 W, 520 W, 585 W, 650 W and 20 kHz) for 30 min, and conventional heating at 30 °C, 40 °C, 50 °C, 60 °C, and 70 °C for 10 min was used for optimizing the dyeing conditions. Following dyeing, the samples were washed in cold water using a liquor ratio of (L: R) 50:2 and a nonionic detergent (laboratory detergent) at a concentration of 3 g/l to eliminate any unfixed dye and were then left to air dry [14].

### 2.11 Liquor-to-fabric ratio

With minor modifications, the spent dye liquor in the cotton, silk and leather dyeing processes was assessed following an earlier study by Sivakumar et al. [4]. At regular intervals, spent dye liquor samples were collected from each dye bath to analyze dye content in cotton, silk, and leather. The % dye exhaustion was calculated using Eq. (2).

(2) $$\%\;\text{Flower petal dye exhaustion}=\frac{\text{Flower petal dye},\;\text{g}-\text{flower petal dye in spent liquor},\;\text{g}\times 100}{\text{Flower petal dye, g}}$$

### 2.12 Color coordination test

The CIE L*, a*, b*, C*, h, and -a* coordinates denote the redness-greenness and yellowness-blueness axes in the color space. The color strength (K/S) of dyed cotton, silk, and leather was assessed using a Data color 600 spectrophotometer from Data Color Company (USA) under illuminant D65 and a 10° standard observer. The test result was calculated as the average of eight readings taken from different positions. The K/S value was calculated using Eq. (3):

(3) $$\text{K}/\text{S}=\left(1-\text{R}\right)2/2\text{R}$$

where *R* is the observed reflectance.

### 2.13 Test for color fastness

Various color fastness tests were performed on dyed cotton, silk, and leather under different dyeing methods. To evaluate the color fastness of the dyed leather samples, ISO 105-C01 was employed for the rubbing fastness test, ISO 105-X12 for the washing fastness test for the dyed silk samples, and ISO 105-B02 for the light fastness test for the dyed cotton fabric samples [4].

### 2.14 Antibacterial activity of the extracted dye

The recommended sterilization procedure was followed (120 °C, 20 min, 15 psi) for all equipment and materials. 5 g of peptone and 3 g of beef extract were dissolved in 1-litre water, and the bacterial culture (*Staphylococcus sp, Pseudomonas* sp, *Vibrio* sp, *Klebsiella* sp, and *Micrococcus* sp) obtained from Bharath Medical College was added to it. The mixture was incubated for 24 h. After autoclaving for 20 min at 121 °C at 15 psi pressure, the medium was poured onto a Petri plate with an equivalent agar layer. The solid culture medium consisted of 5 g of peptone, 3 g of beef extract, and 20 g of agar at a pH of 6.9 ± 0.1. A log-phase bacterial culture of 100 µl was inoculated separately on the agar plates using a spread plate method. Under sterile conditions, filter paper discs measuring 5 mm in diameter were placed on the inoculated agar medium. In the subsequent steps, 10 µl of dye solution was added to each disc individually. Agar plates containing dye discs were incubated at 37 °C for 16 h. The inhibition zone on the plates was examined after incubation [17].

### 2.15 Determination of the minimal inhibitory concentration (MIC) of the dye

The MIC of *C. alata* yellow dye against 5 bacteria was tested with the agar double dilution method. The concentration of the bacteria used in this measurement was about 1 × 10^4^ CFU/mL, and the concentration gradients of *C. alata* yellow dye were 50, 25, 12.5, 6.25, 3.12, 1.56, 0.78 and 0.39 mg/ml. Furthermore, the MIC values of *C. alata* yellow dye against tetracycline were evaluated. In addition, the antibacterial effects of *C. alata* yellow dye under the concentration of 1 MIC or 2 MIC at different time intervals were measured by counting the live bacteria on the plates.

### 2.16 Data analysis

Statistical analysis was conducted using the Statistical Analysis System.

## 3 Results and discussion

Extracting natural dyes from plant sources and dyeing textile materials in a cost-effective, eco-friendly manner while reducing the usage of chemicals, time, energy, and effluent has been achieved by ultrasound for many years. Ultrasound-assisted extraction of polyphenols and flavonoids from *C. tinctoria* Nutt fruit extract has been studied by Zalaru et al. [18], along with their chemical structures. Plant-based pigments or dyes, including betalains, anthocyanins, chlorophylls, and carotenoids, are utilized in various industries such as food additives, textile colorings, livestock feed, pharmaceuticals, cosmetics etc. These dyes are categorized as either fat-soluble or water-soluble. The dye extracted from *C. alata* flowers contains anthraquinones, natural pigments that produce yellow shades, and excellent light fastness and wash fastness properties [19], [20], [21], [22]. Anthraquinones have biomedical applications, such as natural antimicrobial agents, and the extraction parameters influence their quantity and stability. This study aims to optimize the extraction and dyeing potential of *C. alata* flower dye for cotton, silk, and leather. The extracted dye's relative colour strength value was evaluated to determine the optimal conditions for dye extraction and achieve maximum color yield. Various extraction methods produced different levels of color strength based on changes in time, temperature, and solvent.

**Fig. 1:**
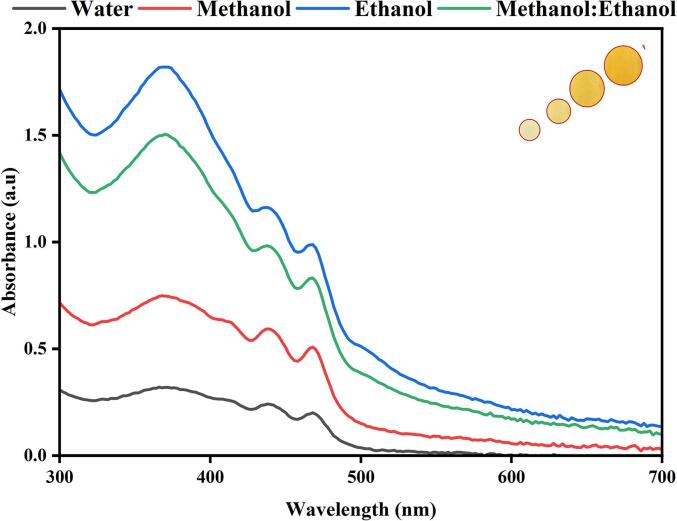
Optimization of *C. alata* dye extraction using magnetic stirrer in different solvents.

**Fig. 2:**
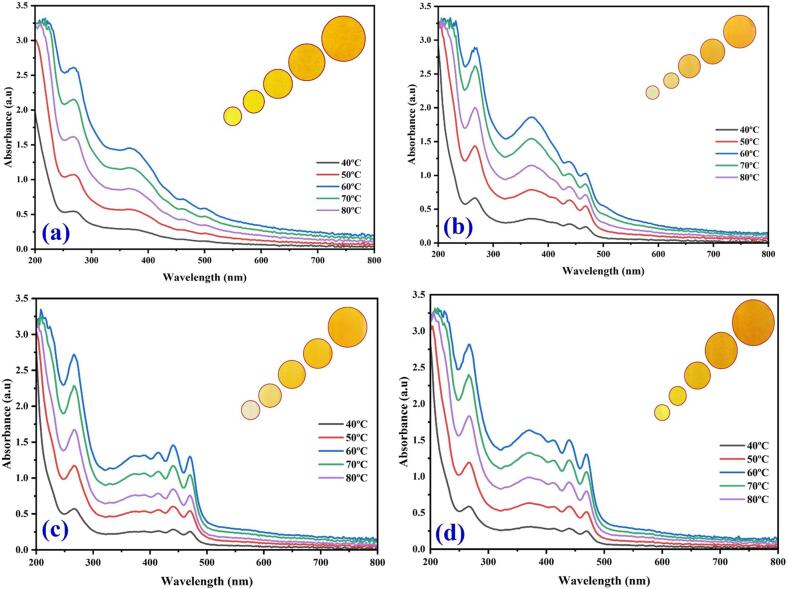
Optimization of *C. alata* dye extraction using conventional heating in different temperatures and solvents (a) aqueous extract, (b) methanol, (c) ethanol, and (d) methanol: ethanol.

**Fig. 3:**
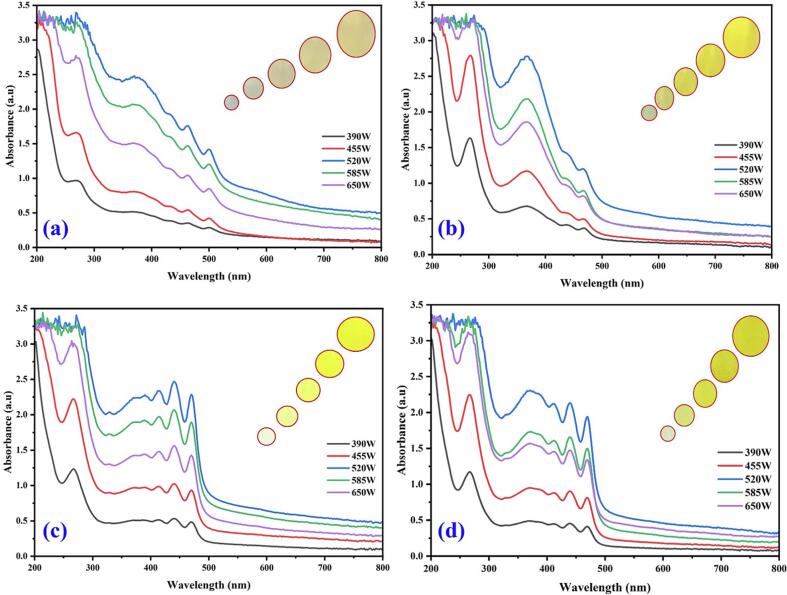
Optimization of *C. alata* dye extraction using an ultrasonic water bath in different temperatures and solvents (a) aqueous extract, (b) methanol, (c) ethanol, and (d) methanol: ethanol.

**Fig. 4:**
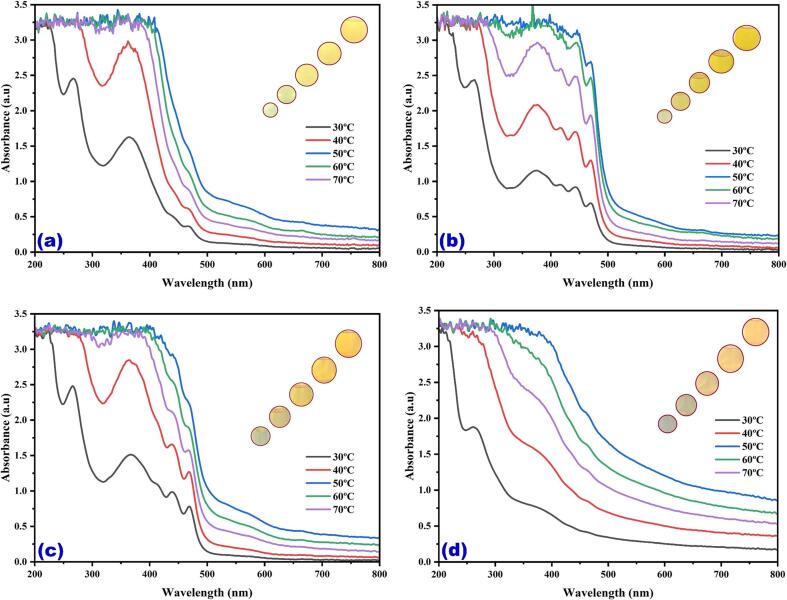
Optimization of *C. alata* dye extraction using an ultrasonic probe in different frequencies (Hz) and solvents (a) aqueous extract, (b) methanol, (c) ethanol, and (d) methanol: ethanol.

In the magnetic stirrer method of extraction, the maximum color intensity was observed in the methanol extraction followed by methanol: ethanol, ethanol and water (Fig. 1). The results indicate that there is a significant improvement in the % yield of coloring matter extract obtained due to the use of continuous agitation which leads to interaction between the solvent and the flower petals. The difference in the enhancement in extraction yield with ultrasound for different plant materials could be due to different degrees of binding of coloring matter attached to plant cell membranes. Fig. 2(a-d) UV–Vis spectral analysis shows the maximum yellow color dye yield in different solvents under the ultrasonic water bath extraction (Fig. 2a water, Fig. 2b methanol, Fig. 2c ethanol, and Fig. 2d methanol: ethanol (1:1), respectively). The optimum dye yield was obtained at 60 °C for 45 min in ultrasonic water bath extraction using 5 g of flower in methanol. The relative color strength values gradually decreased at low temperatures and then increased with temperature and time. The ultrasonic water bath enhanced the colouring component's extraction due to the breakdown of the uppermost layer of the flower by ultrasound, which enhanced the solubility of the coloring component by ionizing hydroxyl (phenoxide) groups in an alkaline medium [23], [24], [25], [26], [27]. Yellow dye exhibited two peaks at 287 and 479 nm, corresponding to anthraquinone. Fig. 3(a-d) shows the UV–visible spectrum of the yellow dye extracted from the flowers of *C. alata* using different solvents using an ultrasonic probe sonicator. Among different power inputs (watts) used for the extraction, 520 W exhibited higher dye yield at 30 min using 5 g of the flower in methanol. Fig. 4(a-d) shows that the higher dye yield could be achieved at 50 °C for 30 min using 5 g of flower employing the direct heat extraction method.

During the direct heat extraction, the dye colour turns brown for an extended duration. However, less time for direct dye extraction leads to lower yield. Hence, the flower is unstable to release the yellow dye into the solvents under low and higher heat. Due to fewer cavitation effects caused by ultrasound in the flower tissue, the dye yield is meager in ultrasonic probe extraction. Due to the uniform ultrasound generated in hot water, the ultrasonic bath augments slowly, releasing the dye into the solvent system. Hence, the stability of the yellow color dye does not change during the extraction. Three different polar solvents and a combination of solvents were used to extract the yellow dye. Methanol alone exhibits excellent dye yield among the three extraction methods. This is due to the high interaction between methanol and dye molecules.

**Fig. 5:**
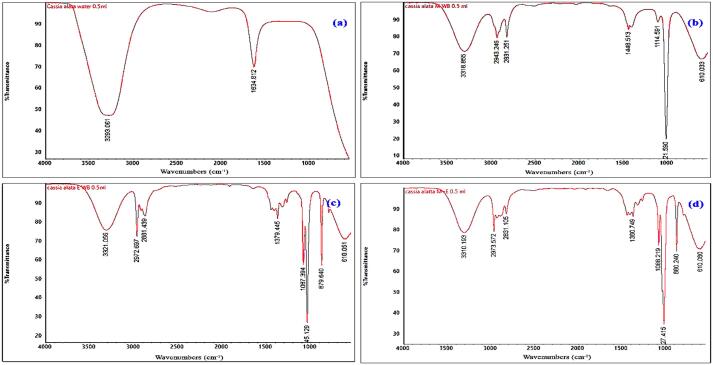
FT-IR spectra of optimized *C. alata* dye extraction using an ultrasonic water bath and various solvents (a) aqueous extract, (b) methanol, (c) ethanol, and (d) methanol: ethanol.

Fig. 5(a-d) shows the FTIR spectra in the spectral range of 4000∼400 cm^−1^ for optimum dye yield using ultrasonic water baths in different solvent systems. Fig. 5(a) shows the spectra of yellow dye obtained by ultrasonically dissolving it in water. The peak at 3293 cm^−1^ is attributed to the hydroxyl stretching of absorbed water, and aromatic C=C double bonds are present at 1634 cm^−1^. Fig. 5(b), the absorption band at 3318 cm^−1^ is due to the O—H stretching of alcohol and water, 2943 cm^−1^ for —C—H aldehydic, 2831 cm^−1^ peak corresponds to the —C—H stretching, 1114 cm^−1^ peak belongs to C—F, 1448 cm^−1^ peak attributed to C=C aromatic, 610 cm^−1^ peak attributed to C—Cl and an unknown peak was observed at 921 cm^−1^. The peaks at 879 cm^−1^ and 1087 cm^−1^ could be assigned to the C—O—C bond of polyphenols (Fig. 5 (c)). The remaining peaks are similar to that of methanol extract (Fig. 5b). Fig. 5(d) peaks between 3310 cm^−1^ ∼ and 610 cm^−1^ for the following methanol and ethanol extract are similar to that of methanol and ethanol alone. The characteristic peaks are similar to those of anthraquinone-containing compounds previously reported [28], [29], [30], [31], [32], [33], [34], [35], [36].

**Fig. 6:**
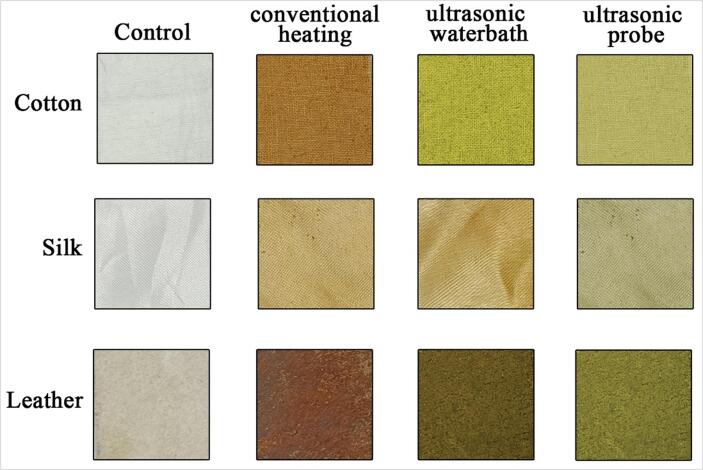
Dyeing optimization of leather, silk, and cotton fabrics dyeing with *C. alata* dye using different techniques.

**Fig. 7:**
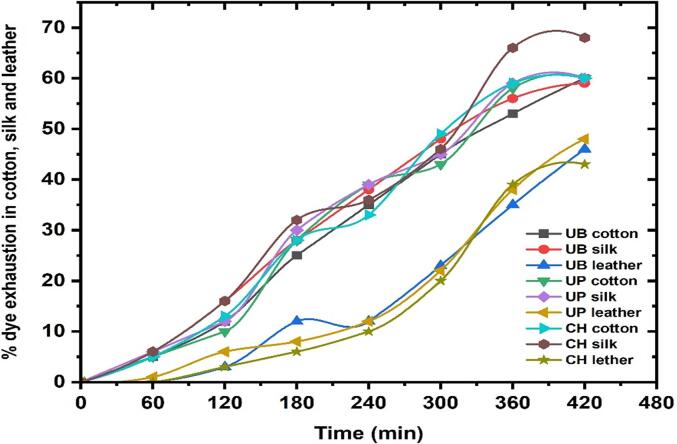
Influence of ultrasonic bath, ultrasonic probe, and conventional heating *C. alata* flower petal dye exhaustion during the cotton, silk and leather dyeing process.

Natural dyes were separated from the *C. alata* flower using a variety of solvent extraction protocols. Degummed, bleached, and surface-cleaned cotton, silk fabrics and leather were treated with the dye extracted from the flower. Fig. 6 and Table 1 show the transparent color, Integ value and colorimetric parameter of dyed cotton, silk and leather under different dyeing methods. The transparent colors of samples 1–4 were yellow–brown, red-brown, brown and dark brown, respectively. The order of samples about Integ value was 1 < 4 < 3 < 2. While dyeing in the direct heating bath, the dye color transformed from yellow to brown in cotton fabric, light yellow shade in silk and dark brown in leather (Fig. 6). This phenomenon is due to the dye molecules oxidising in the dye bath and not able to withstand the direct heating. However, less heating leads to a lower uptake in the dye. Ultrasonic probe dyeing methods lead to spoiling the fabrics and leather under a long exposure time, and exposure for less time leads to poor dyeing. Due to the water in the bath acting as a barrier to control a uniform ultrasound entering the liquor bath, the ultrasonic bath dyeing produced good dyeing without changing the dye colour (Fig. 6). This leads to uniform dye adsorption by the fabric and leather to facilitate the dyeing process without using any moderant. Table 2 presents the results of the color fastness tests of three dyed fabric and leather methods under optimum conditions. After washing twice with boiling soap liquid, the dyed cotton fabric demonstrated good washing and rubbing fastness. However, the color fastness of alkali perspiration scored only 4 (relatively good) due to the pH sensitivity of the color [37], [38], [39], [40], [41], [42], [43]. It was noted that the dark yellow color of the dyed leather, silk, and cotton fabric samples became lighter as the pH increased, which led to a slight decrease in the intensity of the color during alkaline perspiration. Fig. 7 shows the liquor-to-fabric ratio (L: F). The optimum dyeing conditions were 60 °C for the temperature factor, 45 min for the time factor, and 1:10 for the liquor-to-fabric ratio.

**Table 1:** CIE L,a,b values of the dyed leather, silk and cotton fabrics by *C. alata* dye at optimum Condition.

	L*	a*	b*	c*	h◦
**Conventional Heating**					
Cotton	51.62	14.65	36.95	25.14	31.25
Silk	75.54	3.68	39.45	23.84	41.65
Leather	47.53	18.23	28.54	28.56	24.62
**Ultrasonic Water bath**					
Cotton	80.95	−16.52	59.35	10.54	66.5
Silk	78.65	4.35	48.85	20.58	43.85
Leather	61.55	7.35	42.45	23.84	41.65
**Ultrasonic Probe**					
Cotton	88.65	−11.35	55.65	11.34	61.45
Silk	83.45	−1.85	28.75	18.64	48.64
Leather	71.32	−7.54	51.25	15.38	56.43

**Table 2:** Fastness properties of *C. alata* dyed leather, silk and cotton fabrics at optimum condition.

**Ultrasonic Water bath**				
	Rubbing		Wash fastness	Lightfastness
	Dry	Wet		
Cotton	4–5	4–5	4	2
Silk	4	4–5	4–5	3
Leather	3	3	3–4	2
**Ultrasonic Probe**				
	Rubbing		Wash fastness	Lightfastness

	Dry	Wet

Cotton	4	4	3–4	2
Silk	3–4	3–4	4	3
Leather	3	3	4–5	2
**Conventional Heating**				
	Rubbing		Wash fastness	Lightfastness

	Dry	Wet

Cotton	4–5	4	4	2
Silk	4	4	3–4	3
Leather	3	3	4	2

**Fig. 8:**
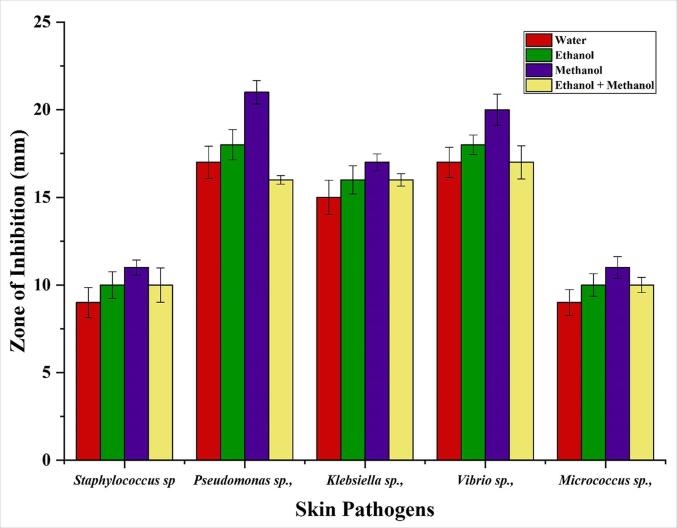
Antibacterial activity of *C. alata* dye against skin pathogens.

In this study, we evaluated the antibacterial properties of the dye obtained from *C. alata* flower against a variety of skin microorganisms (*Staphylococcus sp*, *Pseudomonas* sp, *Vibrio* sp, *Klebsiella* sp, *Micrococcus* sp). The results are shown in Fig. 8. According to the results, the extracted dye was found to have variable antibacterial activity against the pathogens tested. In contrast, the dye extraction using methanol had excellent activity against all the pathogens. At the same time, the other solvents, like water, ethanol, and ethanol: methanol exhibited limited activity (Fig. 8). Additionally, the study evaluated the dye's minimum inhibitory concentration (MIC) against the tested pathogens and found it highly effective. A positive control, tetracycline, had a MIC of 25 µg/ml, as shown in Table 3. The zone of inhibition increases with increasing dye concentration, which interacts with the bacterial cell membrane, binds to the organelles of the mesosome, reduces mesosomal function, and increases the generation of reactive oxygen species (ROS) in the mesosome [44], [45].

**Table 3:** Minimum inhibitory Concentration of *C. alata* extracted dye against skin pathogens.

**Pathogens**	**MIC (mg/ml)**	**Tetracycline (µg/ml)**
*Staphylococcus* sp,	12.5	0.39
*Pseudomonas* sp,	1.56	0.39
*Klebsiella* sp,	3.12	0.39
*Vibrio* sp,	3.12	0.39
*Micrococcus* sp	25.0	0.78

## 4 Conclusion

The extraction efficiency of colorant from *C alata* flower petals is shown to have significantly improved with the application of ultrasound, as first reported. Using 45 W of ultrasonic power for 30 min, it has been found that a 1:1 ethanol-methanol mixture yielded higher yields and extraction efficiency. Three different extraction processes have been used, and it has been revealed that they help increase the production of the *C. alata* flower petal yellow dye. Out of three extraction protocols, the ultrasonic water bath augments 5% dye production and uses 45 W using 1:1 ethanol-methanol. Using plant materials like *C. alata* flower petals, this strategy can also extract colors from them for analytical purposes. The extracted *C. alata* The capacity of flower petal dye for coloring. It is excellent for dyeing cotton, silk, and leather.

Additionally, natural dyeing of cotton, silk, and leather is advantageous with an improved exhaustion rate by ultrasound. The mechanism for the improvement in ultrasound extraction could be the rupturing of the flower petal cell wall and the release and better transport of the *C. alata* flower petal dye into the external medium, mediated by micro stirring and acoustic streaming effects generated by ultrasound cavitation. The current work so distinctly provides a better extraction process from natural dye resources, such as the flower petal of the *C. alata*, even when external heating is not required, and for the dyeing of cotton, silk, and leather. The yellow flower petal dye from the *C. alata* plant may be necessary for applications involving sensitive medical issues. Thus, soon, in light of increased environmental concerns, eco-friendly, non-toxic dyeing of fiber materials could be a feasible “Green chemistry“ alternative for dyeing companies.

## CRediT authorship contribution statement

**Moorthy Muruganandham:** Methodology, Conceptualization. **Kanagasabapathy Sivasubramanian:** Writing – original draft, Writing – review & editing. **Palanivel Velmurugan:** Investigation, Writing – original draft, Writing – review & editing. **Subbaiah Suresh Kumar:** Investigation, Writing – original draft, Writing – review & editing. **Natarajan Arumugam:** Visualization. **Abdulrahman I. Almansour:** Project administration, Resources, Software. **Raju Suresh Kumar:** Funding acquisition. **Sivakumar Manickam:** . **Cheng Heng Pang:** . **Subpiramaniyam Sivakumar:** Formal analysis, Supervision.

## Declaration of Competing Interest

The authors declare that they have no known competing financial interests or personal relationships that could have appeared to influence the work reported in this paper.
